# Hybrid Approach to Complex Stanford Type B Dissection: Unusual Extra-anatomical Bypass (Aorto-Celiac-Mesenteric Bypass) + Thoracic Endovascular Aortic Repair (TEVAR) + Cholecystectomy

**DOI:** 10.21470/1678-9741-2020-0123

**Published:** 2021

**Authors:** Sinan Demirtaş, Nazım Kankılıç, Celal Yavuz

**Affiliations:** 1 Department of Cardiovascular Surgery, Özel Bower Hospital, Diyarbakir, Turkey.; 2 Department of Cardiovascular Surgery, Medical School of Harran University, Sanliurfa, Turkey.; 3 Department of Cardiovascular Surgery, Medical School of Dicle University, Diyarbakir, Turkey.

**Keywords:** Endovascular Procedures/Stents, Except PCI, Extra Anatomical Bypass, Aortic Dissection (Incl Ulcers, Hematomas), TEVAR

## Abstract

Patients with complex Stanford type B aortic dissection are very difficult to treat. Many methods have been proposed so far in the treatment of these patients, and the emergence of hybrid techniques has made the treatment easier. In this article, we shared the extra-anatomical bypass (aorto-celiac-mesenteric bypass) + thoracic endovascular aortic repair + cholecystectomy operation technique applied to a patient with complex type B aortic dissection.

**Table:** 

Abbreviations, acronyms & symbols	
**CA**	**= Celiac artery**
**FL**	**= False lumen**
**RA**	**= Renal arteries**
**SMA**	**= Superior mesenteric artery**
**TEVAR**	**= Thoracic endovascular aortic repair**
**TL**	**= True lumen**

## INTRODUCTION

Untreated aortic aneurysms and dissections are diseases that may lead to morbidity and mortality. Lately, hybrid treatments have been presented as alternative ways of solution for patients with high risk and complex aortic aneurysm^[^^1^^-^^4^^]^. These hybrid attempts prevail cardiopulmonary bypass and reduce the risks that may be caused by it^[^^5^^]^. In type 3 aortic dissections, thoracic endovascular aortic repair (TEVAR) surgery can be safely performed. However, a hybrid approach can be a necessity when the TEVAR procedure is not sufficient on its own. In this paper, we shared the extra-anatomical bypass (aorto-celiac-mesenteric bypass) + TEVAR + cholecystectomy approach that had never been performed before.

## HOW I DO IT

**Fig. 1:**
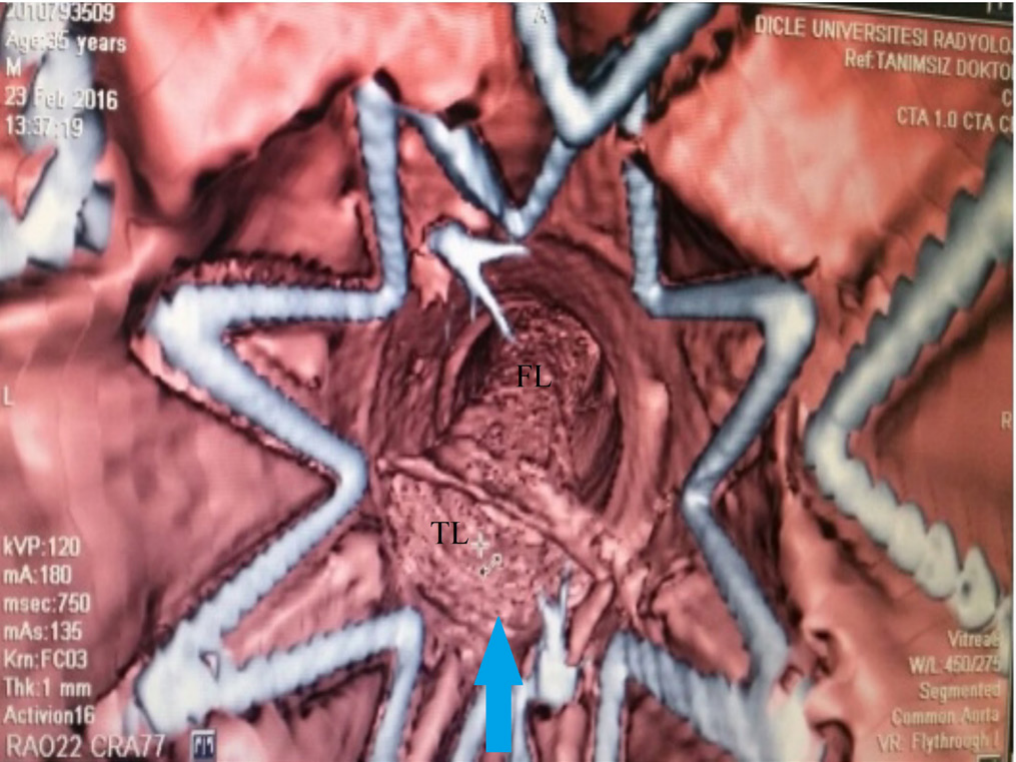
tomography scan, display of the true lumen (TL) and false lumen (FL). TL is seen to narrow (stent graft-induced new entry, or SINE).

**Fig. 2:**
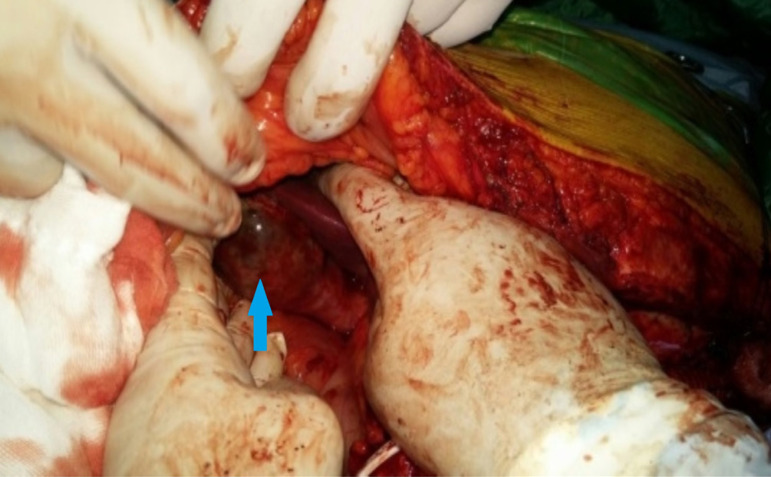
Ischemic hydropic gallbladder with necrosis.

**Fig. 3:**
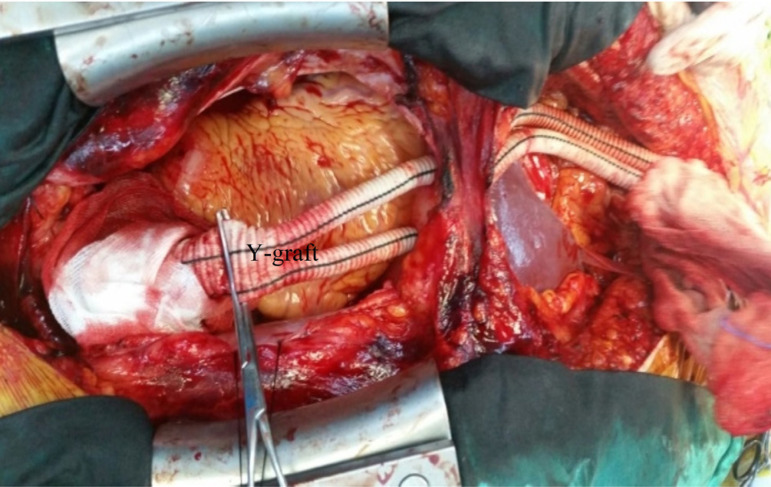
Passage of vascular Y-graft from mediastinum to abdominal cavity.

**Fig. 4:**
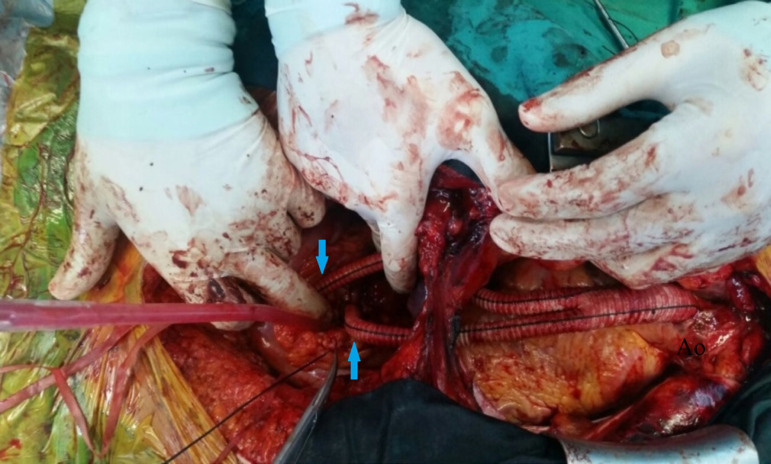
Vascular Y-graft from ascending aorta to superior mesenteric artery and celiac artery after anastomoses.

**Fig. 5:**
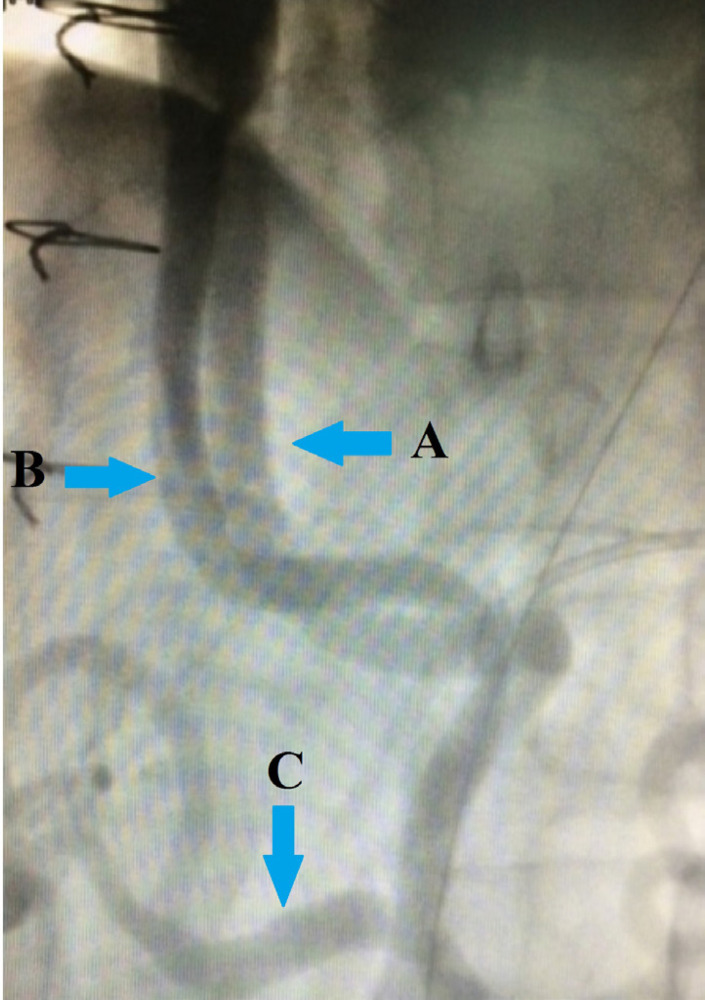
Angiographic view of Y-graft which perfused superior mesenteric artery and celiac artery. A) Dacron Y-graft from the ascending aorta to the celiac artery; B) Dacron Y-graft from the ascending aorta to the superior mesenteric artery; C) celiac artery branches.

**Fig. 6:**
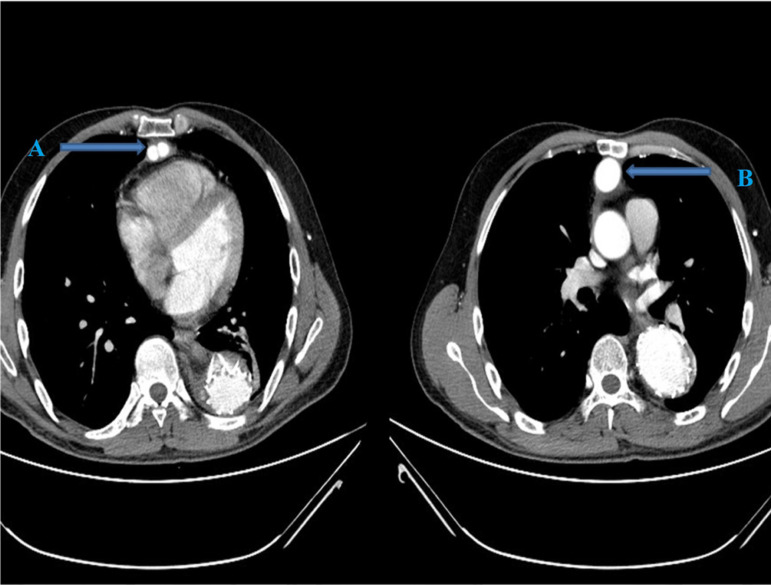
Follow-up computerized tomography angiography image at the first year. A) Distal legs of Y-graft are anastomosed to celiac and superior mesenteric arteries; B) proximal segment of Y-graft anastomosed to ascending aorta.

A 36-year-old male patient was referred to our department with acute Stanford type B aortic dissection diagnosis. The patient had no additional comorbid condition, except for hypertension. The aortic tear started immediately distal (aortic isthmus) to the left subclavian artery. The distal part of the tear extended to the femoral arteries. The proximal part extended to the subclavian artery with retrograde flow. Celiac artery (CA), superior mesenteric artery (SMA), and renal arteries (RA) were branching from the true lumen. The patient had ongoing back pain and leg pain refractory to medical treatment. TEVAR procedure (Valiant Thoracic Stent Graft System, Medtronic Vascular, Santa Rosa, California, United States of America) (44 x 44 x 150 mm) was applied to the patient. During the procedure, the left subclavian artery orifice was occluded. Postoperative ischemia was not observed. On the 5^th^ day following the procedure, the patient had growing complaints of stomachache, nausea, and loss of appetite. On the reapplied tomography scan, a new rupture in the distal zone of the placed stent was detected (stent graft-induced new entry, or SINE) (Figure 1). True lumen was observed to narrow (Figure 1). Surgical intervention was decided because of a new tear at the distal end of the graft stent leading to ischemia. Under general anesthesia, the mediastinum was opened together with the upper abdomen with median sternotomy and upper belly median incision. CA and SMA were explored. A cholecystectomy was applied after the exposure of necrotic areas on hydropic gallbladder (Figure 2). By placing side clamp on the ascending aorta, an end-to-side proximal anastomosis was applied using 16 x 8 mm Dacron Y-graft (Figure 3). Y-graft legs were passed to the abdominal cavity through a canal formed in pericardium and diaphragm. The distal ends of the graft legs were anastomosed to SMA and CA (Figure 4), which were ligated at the point they arise from aorta. Thoracic endovascular stent graft (Valiant Thoracic Stent Graft System, Medtronic Vascular, Santa Rosa, California, United States of America) (44 x 44 x 200 mm) implantation was performed through the left femoral artery so as to cover the ostia of SMA and CA. The proximal segment of the endovascular stent graft was placed inside the previous stent graft. Angiography revealed that the Dacron Y-graft was functioning well and delivering blood to CA and SMA. Also, it confirmed the perfusion of RA through the true lumen (Figure 5). Hybrid intervention was ended by duly closing the mediastinum and abdominal wall. Follow-up computerized tomography angiography was performed at the first year, which revealed that SMA and CA were perfused via the Y-graft (Figure 6).

## DISCUSSION

In the last decade, hybrid operations have been introduced in daily clinical practice of cardiovascular surgery in the management of patients presented with complex aortic aneurysm and dissections. In a study with 172 patients having complex aortic arch pathologies, thoracic endovascular grafting integrated to traditional supra-aortic arch debranching bypasses was evaluated in high-risk patients. It was concluded that hybrid approach in aortic arch repairment can be an alternative to open surgical procedures in high-risk patients and emergency cases^[^^3^^]^. Yokoyama et al.^[^^6^^]^ evaluated the two-step repairment and hybrid treatment in patients with distal aortic arch aneurysm and suggested that they are both appropriate methods to avoid paraplegia complication. In another study with a follow-up period of 18 months, the use of hybrid strategies was studied in a total of 31 patients who were not eligible for endovascular treatment. The authors stated that this combined approach would expand the potential area of endovascular grafting usage, which can be an appropriate way of solution in patients with poor cardiopulmonary reserve^[^^4^^]^. In a recently published review, open surgery, endovascular intervention, and hybrid treatment choices were evaluated in patients with type B dissections. It was reported that high-risk patients without complications could benefit from endovascular repairment, and emergency thoracic endografting is considered as the gold standard method for all cases with complex type B dissections. Also, hybrid approach was emphasized as a reasonable treatment option in high-risk patients for open surgery^[^^7^^]^.

Hybrid prosthetics staffs have significantly contributed to various surgical procedures^[^^8^^]^. Post-endovascular procedure complication as a new aortic tear distal to endovascular graft developed in our patient and was treated with a vascular Y-graft and endovascular graft stent as a new anti-dissection hybrid approach.

Similar to our technique, perfusion of renal arteries could be achieved through the iliac arteries in a retrograde way with the transperitoneal approach. However, the same situation will be very difficult to perform in CA and SMA. This will be more difficult than the technique we apply. Also, providing CA and SMA perfusion from the proximal segment without dissection will reduce the problems that may occur afterwards.

## CONCLUSION

We think that extra-anatomical bypass + TEVAR procedure can be considered as an alternative surgical technique in patients with complex Stanford type B aortic dissection that can be safely performed to avoid organ ischemia.

**Table:** 

Authors' roles & responsibilities	
SD	Substantial contributions to the conception or design of the work; or the acquisition, analysis, or interpretation of data for the work; final approval of the version to be published
NK	Substantial contributions to the conception or design of the work; or the acquisition, analysis, or interpretation of data for the work; final approval of the version to be published
CY	Substantial contributions to the conception or design of the work; or the acquisition, analysis, or interpretation of data for the work; final approval of the version to be published
